# Two Case Reports of Benign Testicular Mesothelioma and Review of the Literature

**DOI:** 10.1155/2017/5419635

**Published:** 2017-01-12

**Authors:** Cristobal Ramirez Sevilla, Carme Admella Salvador, Josep Feliu Canaleta, Juan Llopis Manzanera, Miguel Angel Barranco Sanz, Juan Antoni Romero Martin, Sergi Bernal Salguero

**Affiliations:** Mataro Hospital, Barcelona, Spain

## Abstract

Mesothelioma is usually diagnosed in people over the age of 50 with large history of asbestos-related exposure. It is frequently located in pleural cavity, peritoneum, and pericardium. At the testicles the mesothelioma had been reported first in 1957 like a malignant non-germ-cells tumor. The objective is to present two case reports of benign testicular mesothelioma and review of the literature.

## 1. Introduction

Mesothelioma is usually diagnosed in people over the age of 50 with large history of asbestos-related exposure. It is frequently located in pleural cavity, peritoneum, and pericardium. At the testicles the mesothelioma had been reported first in 1957 by Bárbera and Rubino [1] like a malignant non-germ-cells tumor.

## 2. Objective

The objective of this paper is to present two case reports of benign testicular mesothelioma and review of the literature.

## 3. Case Report 1

**Figure 1:**
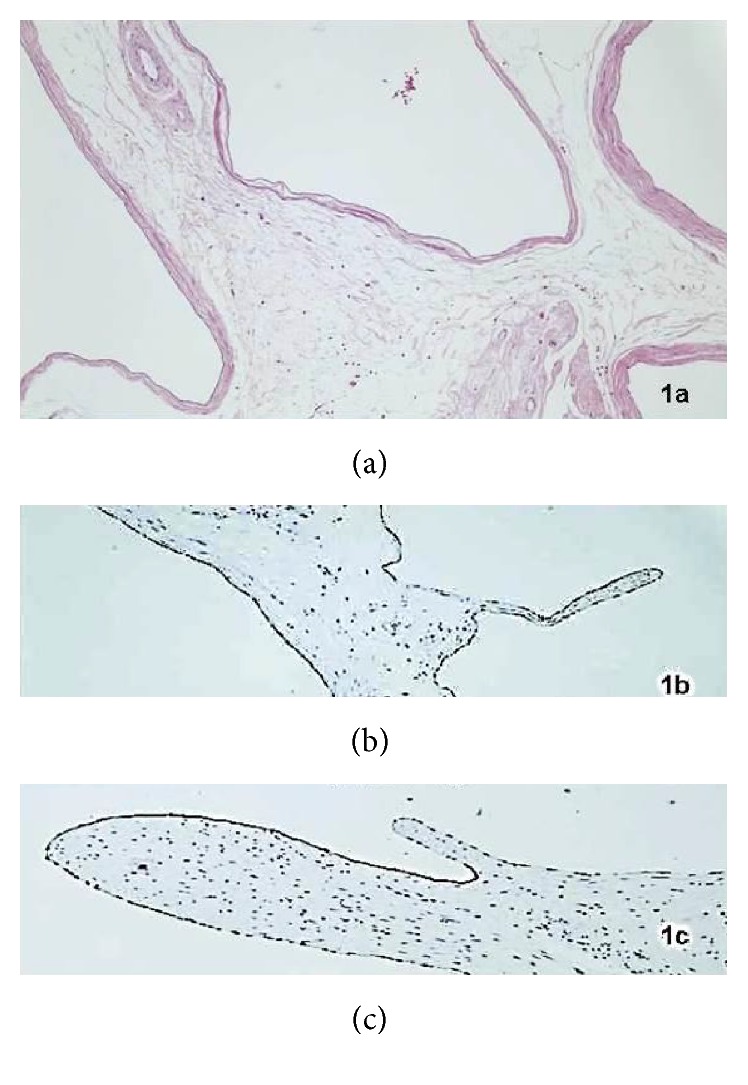
(a) Cyst cavities with single epithelium. (b) Calretinin +. (c) Epithelial membrane antigen + (EMA).

A 74-year-old patient with history of alcoholic liver disease, type 2 diabetes mellitus, hypertension, peripheral vascular disease, and depression visited the urology department with a left inguinoscrotal hernia and left testicular atrophy confirmed by physical examination and ultrasound. A Lichtenstein surgical technique was performed with inguinal orchiectomy. The pathologist indicated the presence of saccular proliferation in the wall of the hernia with cysts and simple epithelium without stromal invasion (Figure 1(a)). Immunochemistry was positive for calretinin (Figure 1(b)), epithelial membrane antigen (Figure 1(c)), and AE1 and AE3 cytokeratins. The final diagnosis was benign cyst mesothelioma of the peritoneum and testicular atrophy. Abdominal CT scan showed absence of distant disease. There is no evidence of disease 10 years after surgical treatment.

## 4. Case Report 2

**Figure 2:**
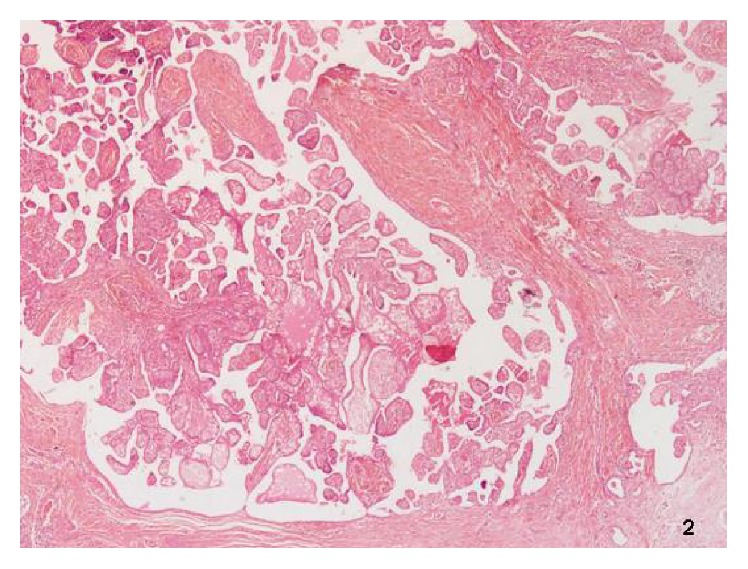
Papillary structure with inflammatory cells.

A 32-year-old patient born in Senegal without history of disease visited the emergency department because of an augmentation of the left testicle without symptoms. Physical examination and ultrasound showed a hydrocele with 16 × 12 cm of length. Surgical treatment was performed by testicular approach and pathologist indicated the presence of yellowish exophytic 2,7 × 2,2 cm mass. The microscopy identified a proliferation depending on tunica vaginalis with papillary architecture and inflammatory cells, fibroblasts, and foaming histiocytes with psammoma bodies, without atypia, mitosis, and vascular invasion (Figure 2). Immunochemistry was positive for large spectrum cytokeratins and vimentin and negative for CEA and VII factor. The final diagnosis was well-differentiated papillary mesothelioma of the tunica vaginalis in the wall of the hydrocele. CT scan of the chest, abdomen, and pelvis did not show distant disease. 21 years after the diagnosis, the patient is free of disease.

## 5. Discussion

Mesothelioma is caused by a mutation of mesothelial cells that make up the lining of organs as lung, pericardium, peritoneum, and testicles. The lining of the testicles is known as the tunica vaginalis. When this tumor develops in the tunica vaginalis, the lining starts to thicken and produce fluid buildup. The testicular mesothelioma can be classified in epithelial tissue, cystic and mixed. The papillary structure of this tumor is more frequent in the tunica vaginalis [2–4].

Testicular mesothelioma appears frequently in patients with history of hydrocele, inguinal hernia, and paratesticular mass [5–7]. The most common symptom of testicular mesothelioma is the swelling of the testicles, but it is not specific. Moreover, the mesothelioma has a long latency period that makes it hard to diagnose. Some cases of testicular mesothelioma have been associated with asbestos exposure but there is a lack of research on this localization of mesothelioma.

The preoperative diagnosis is unusual; however the testicular mesothelioma is frequently an aggressive cancer. It has more than 50% of local or distant recurrences.

Mesothelioma in the lining of the testicles is a rare form of an extremely rare cancer. It typically develops over the age of fifty. Predisposing factors are testicular trauma, large history of hydrocele, or inguinoscrotal hernia. Life expectancy for pleural mesothelioma is worse than testicular mesothelioma.

In contrast with malignant testicular mesothelioma there are well-differentiated cases [8–11]. The pathological criteria of benignity for well-differentiated testicular mesothelioma are mitotically inactive, no evidence of stromal invasion, no vascular invasion, absence of atypia, low lymphocyte infiltration, and immunochemistry positive for epithelial membrane antigen and calretinin [2–4].

The two cases reported are well-differentiated with good prognosis, one younger than the average age.

Preoperative diagnosis of benign testicular mesothelioma is difficult. When intraoperative biopsy is performed and benignity is confirmed, local excision rather than orchiectomy is recommended [2].
